# Augmented Reality for Anatomy Training: Efficacy and Interest Among Medical Trainees

**DOI:** 10.1089/jmxr.2024.0006

**Published:** 2024-10-18

**Authors:** Matthew K. Mitchell, Alexander W. Burns, Sohail Mohammed, Eleni Balasalle, Blaire K. Rikard, Samir Ghandour, Laura Avery, Min Lang, Raul Uppot

**Affiliations:** 1 Harvard Medical School, Boston, Massachusetts, USA.; 2 Department of Radiology, Massachusetts General Hospital, Boston, Massachusetts, USA.; 3 Department of Radiology, Brigham and Women's Hospital, Boston, Massachusetts, USA.

**Keywords:** augmented reality, education, medicine, technology, medical extended reality

## Abstract

Augmented reality (AR) technology has steadily advanced since its inception, resulting in serious discussion of its utility in various fields, including medical education and training. In an attempt to address these questions, we conducted a prospective study at a U.S. medical school using an AR-based anatomy education software. Medical students in their preclinical years were invited to participate in the prospective study. Participants completed a presurvey that characterized familiarity with and interest in AR. Volunteers then engaged with three modules within the AR-based anatomy education software. After finishing the modules, participants completed a postsurvey regarding their experiences with and perceptions about AR. This study identified that less than half of medical students surveyed felt familiar with AR technology and its potential role within medical education prior to study exposure. A statistically significant difference was demonstrated between students’ perceptions toward the use of AR in medical education prior to guided use when compared to students’ perceptions after study exposure (*p* < 0.001). Users’ sentiments, as gauged by postsurvey free responses, were largely in favor of AR as a supplement to existing wet lab dissections rather than as an outright replacement. This study demonstrated a potential use case for AR technology in the supplementation of traditional medical education methods.

## Introduction

Augmented reality (AR) is a technology that integrates real-world environments with computer-generated images in order to create an immersive, hybrid reality. Only recently has the technology advanced to the point of making stand-alone headsets a possibility for the individual consumer. In 2013, Google launched its first attempt at AR with Google Glass.^1^ Later, in 2016, Microsoft announced its first AR headset, HoloLens.^2^

Although AR has wide-ranging applications, one of AR’s most promising frontiers is its use in viewing and interacting with three-dimensional (3D) models. Prior to its development, virtual models were confined to a flat, two-dimensional computer screen. With the implementation of AR, models were transformed from 2D to 3D. AR-enabled users to step into a virtual space and interact with such models in real time, unlike anything that had been done before. This advancement opened the door to real-world applications in countless industries, with companies such as Volkswagen (Wolfsberg, Germany) using AR models in their product design and development, resulting in improved design accuracy and increased quality assurance.^3^

In as much as AR is a powerful and emerging technology, so too is virtual reality (VR). The distinction between AR and VR is best illustrated by comparing headsets with the power off. A VR headset without power essentially blindfolds users from their physical surroundings, limiting interactions with these spaces, while an AR headset allows users to still see and navigate their environment seamlessly. Consequently, AR offers potentially greater versatility as it allows users to train in the same physical space where they will practice and gives them real tools to obtain tactile feedback and build psychomotor skills.^4^ In comparison to VR, AR has also been reported to be more comfortable due to reduced dizziness and headaches as the user’s vision of the real world is unhindered.^5^ To bridge this gap, pass-through technology or mixed reality uses cameras and sensors to overlay a fully digital projection of the real world and virtual elements inside the headset. AR’s success, combined with its greater accessibility and better functionality, has made it a topic of significant discussion within medical education. Used in conjunction with tools such as in-person, hybrid, and remote learning, AR systems offer improved continuity of education in the face of a dynamic learning environment, providing unfettered access to resources 24 h a day.

Specifically, AR has the power to transform the acquisition of anatomical knowledge within the field of medical education. Traditionally, cadaveric dissection has served as the cornerstone of the anatomy curriculum within medical school. Yet, this teaching approach has limitations with regard to cost, physical space, maintenance, and access. According to Kamphuis et al., AR has the potential to circumvent these limitations by creating a “meaningful situated learning experience that may enable the transfer of learning into the workplace.”^4^ As medical schools work on mirroring modern-day medical practice, small-group collaboration has taken a central role in many top training programs.^6^ Ultimately, AR technology allows users to learn from anywhere in the world within a shared virtual space.

Several institutions have already adopted the AR modality in place of traditional dissections-based anatomy. Namely, Case Western Reserve University School of Medicine (CWRU SOM) designed a program deemed HoloAnatomy, a 3D software suite that gives students access to virtual models of every part of the body. Although initial studies have been performed on the efficacy of similar programs, limited data exists.^7^

Hence, this prospective cohort study seeks to build off of the initial HoloAnatomy studies in order to better understand the role of AR in the training of medical students. In this article, a complete assessment of students’ perceptions before and after exposure to an AR anatomy experience is provided.

## Materials and Methods

### Study design and participants

This study was conducted at an established, U.S.-based medical school, in collaboration with one of their affiliate hospital’s Department of Radiology. This prospective survey study was approved by the Institutional Review Board with a waiver of informed consent, and permission was obtained from the study institution’s Medical School Dean and the Radiology Clerkship director. Participant privacy was ensured in compliance with the Family Educational Rights and Privacy Act.

This study took place over a 3-week period from September 2021 to October 2021. Invitations to participate were extended to students in their preclinical years. Total participants (*n* = 21) included first-year medical or dental students (*n* = 3) at the beginning of their first academic year and second-year medical or dental students after the completion of their first academic year and immediately before their core clerkship clinical rotations (*n* = 18). Participation was voluntary and verbal consent was obtained for all participants.

### Hardware and software

Four HoloLens 2 AR headsets (Microsoft, Redmond, WA) were used for the study. Each of these headsets was preinstalled with HoloAnatomy (a proprietary educational software developed by Case Western Reserve University, Cleveland, OH).

Three modules were used, consisting of the vascular, gastrointestinal, and cardiac systems.

Two participant roles exist within HoloAnatomy: (1) teacher and (2) student.

1. Teacher
  a. The teacher is the person who begins the anatomy session and controls the various instructional tools, which include:
    - Magnify—places a circular ring around the main anatomical structure, which one can move over different parts to view a magnified version displayed on the side of the model.
    - Pointer—places a small white dot to show students where the teacher is looking.
    - Slide controls (forward and backward)—allow the teacher to go to the next or previous model in the given module.
2. Student
  b. The students log into a collaborative, synchronized anatomy session hosted by the teacher. All students were located in the same physical space (a large, open classroom). The students and the teacher move around the same holographic model simultaneously, with the teacher in control.

All users, in groups of two to three at a time, were logged into the same model simultaneously. In each module, one person functioned as the teacher.

### Study protocol

The study protocol consisted of three phases: orientation, participation, and reflection.

### Orientation

Participants had 5 min to read the protocol first. Afterward, prestudy surveys were administered in the form of a quick response code (QR) code that participants scanned and completed with their smartphones. Tablets and hard copies were available for those without access to an internet-compatible smartphone. All survey responses were de-identified.

**FIG. 1.**
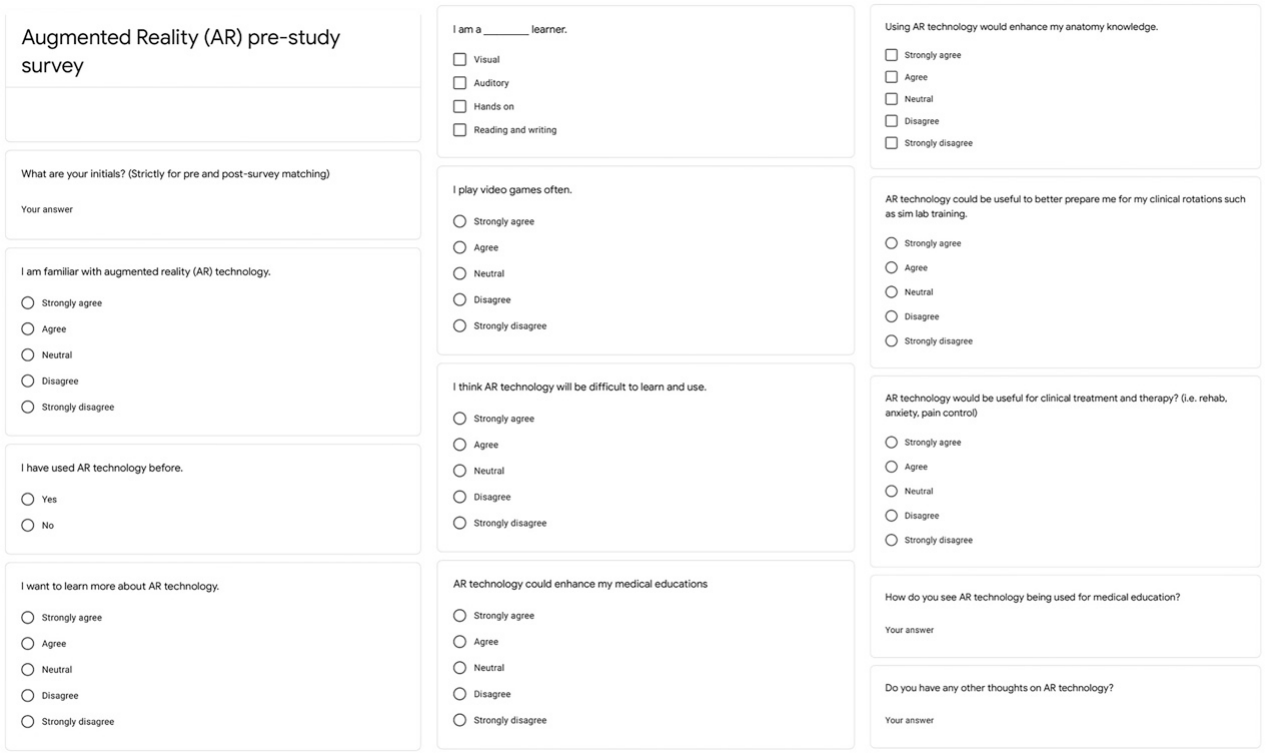
Prestudy survey questions.

The presurvey consisted of 13 Likert-style questions aimed at assessing participants’ background, interest, and familiarity with AR. Questions were answered on a 5-point Likert scale: strongly agree, agree, neutral, disagree, and strongly disagree (Fig. 1).

**FIG. 2.**
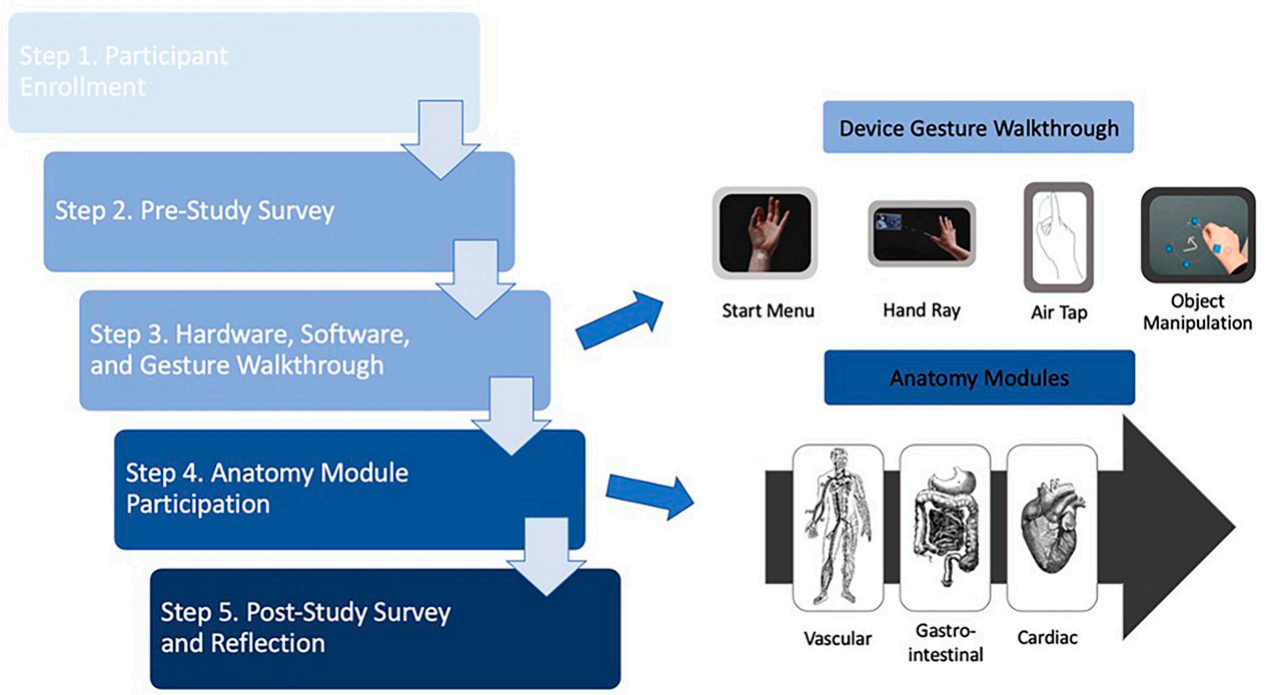
Study protocol.

Participants were subsequently taught the basic gestures and functions that would be used throughout their experience via a visual presentation displayed on the central TV screen (Fig. 2).

### Participation

Users were verbally guided to the Windows Start menu and asked to launch a preinstalled tutorial program called “Tips.” Participants then completed the tutorial module labeled “Hands.” These hands-on exercises familiarized participants with the essential functions of the hardware and hand gestures (Fig. 2). Participants were allowed to progress through this part of the tutorial at their own pace, though no participant took longer than 15 min. Users were subsequently instructed to launch the HoloAnatomy program. All participants joined the same AR anatomy session, taking turns as the “teacher” for each module in order to gain familiarity with the various tools the session instructor had at their disposal. Users completed all slides in three modules—beginning with “General Vascular Anatomy,” then “Gastrointestinal,” and finally “Cardiac.” Participants were given 30 min to 1 h to finish all sessions, which were self-paced. Thereafter, the units were cleaned with antimicrobial cloths between uses.

### Reflection

For the reflection phase, a poststudy survey was administered under the same conditions as the presurvey—users scanned a provided QR code using their smartphones, or the user filled out a hard copy.

**FIG. 3.**
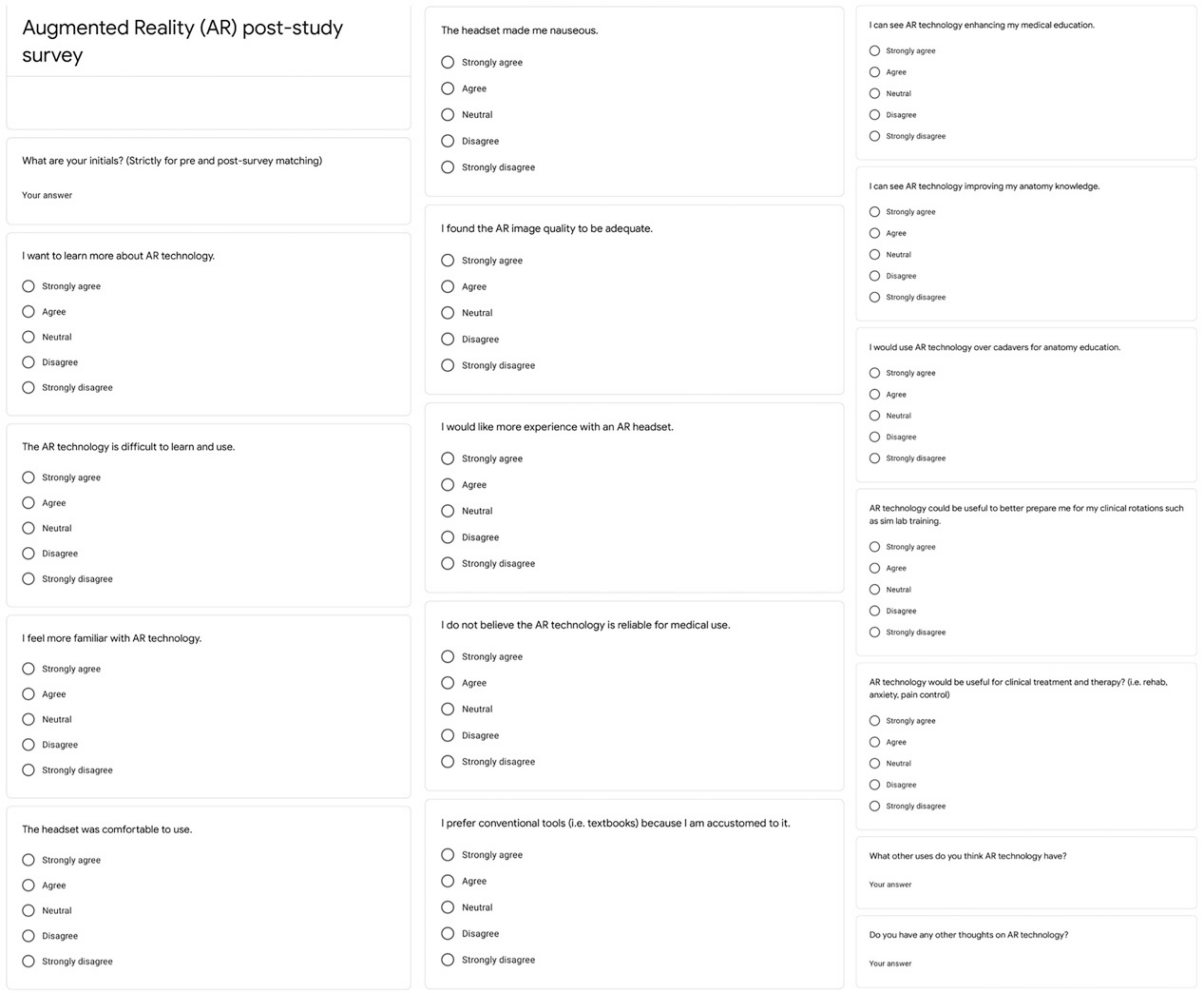
Poststudy survey questions.

The postsurvey consisted of 17 Likert-style questions aimed at assessing study objectives. Questions were answered on a 5-point Likert scale: strongly agree, agree, neutral, disagree, and strongly disagree (Fig. 3). All participants completed the poststudy survey. Question 9 was determined in retrospect to contain biasing language and as such, was omitted from the analysis.

### Statistical analysis

For the four prestudy survey questions that corresponded to the poststudy survey questions, a two-sample *T*-test was performed to determine if there was a difference in the mean before vs. after participation. Specifically, a *T*-test was performed comparing prestudy survey questions 7, 8, 9, and 10 (Fig. 1), which corresponded sequentially to poststudy survey questions 10, 11, 13, and 14 (Fig. 3). A significance level of α = 0.05 was used. The null hypothesis was defined as the difference in the means being due to random chance. The alternative hypothesis was defined as a statistically significant difference between the means.

## Results

### Study population demographics

Every respondent self-characterized as either a visual and/or hands-on learner (100%). The majority identified as both visual and hands-on learners (57%). Sixty-two percent of participants had used AR in some capacity prior to participation, with 48% describing themselves as familiar with AR. Twenty-five percent identified with the statement, “I play video games often.” The median opinion of participants prior to the study intervention regarding the perceived difficulty of learning AR was found to lie within the “neutral” category (Table 1).

**Table 1.** Study Population Demographics

Statement	Positive	Neutral	Negative	Mode	Median
	***n* (%)**	***n* (%)**	***n* (%)**		
1. I am familiar with augmented reality (AR) technology.	10 (48)	1 (5)	10 (48)	4	3
2. I have used AR technology before.^a^	13 (62)	—	8 (38)	—	—
3. I want to learn more about AR technology.	18 (86)	3 (14)	0	4	4
5. I play video games often.	5 (25)	3 (15)	12 (60)	2	2
6. I think AR technology will be difficult to learn and use.	5 (24)	9 (43)	7 (33)	3	3

Median and mode were calculated using 1 = strongly disagree, 2 = disagree, 3 = neutral, 4 = agree, 5 = strongly agree.

a Yes or no question.

### Written responses

**FIG. 4.**
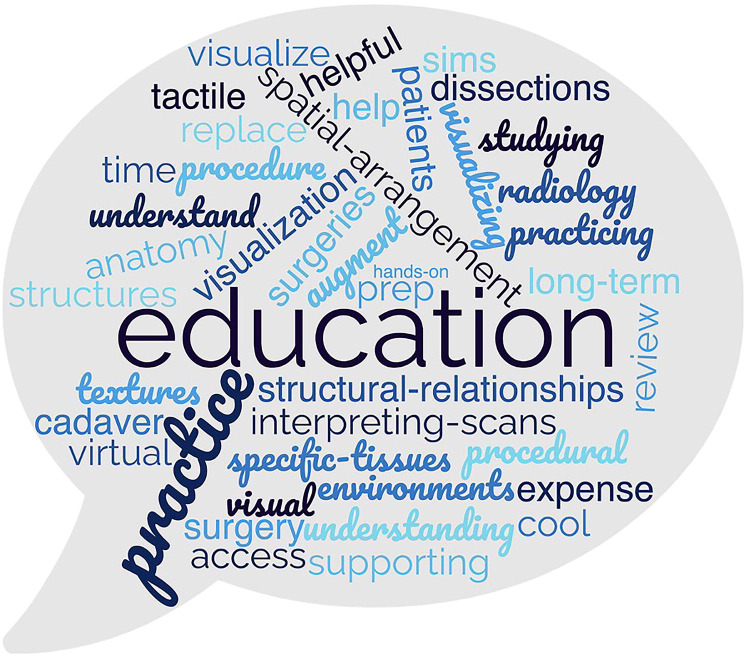
WordCloud depicting responses from pre- and postsurvey free-response questions.

Common themes among respondents’ free-text answers associated with the use of AR technology were “education,” “practice,” and “helpful” (Fig. 4).

### Postparticipation findings

After the intervention, 90% of users expressed enjoyment of the technology. Three users (14%) did, however, report sensations of nausea—a common complaint with AR/VR headsets. Of these users who experienced nausea, two still expressed a desire to learn more about the technology and one felt neutral about this statement. Still, 90% of participants reported that their experience resulted in a desire to learn more about AR technology.

### Usefulness in anatomy curricula

Many said the technology could be useful in learning anatomy but felt it should play a supportive role in existing dissection labs, rather than a primary role. Few felt that AR could, or should, replace conventional dissection labs altogether. When asked if AR could improve their education, the mode of responses was “*strongly agree,”* and the median was *“agree.”* When asked if they would use AR over traditional anatomy labs, both the mode and median of responses was “*disagree.”* To that end, many participants cited the absence of tactile feedback and the occasional clunkiness of the HoloAnatomy interface as prohibiting factors to wet lab replacement (24%).

### Usefulness in medical education

Participants felt this technology would be useful in skill development within surgical and procedural fields. Several participants proposed the concept of projecting real patient scans and images intraoperatively to assist in surgeries.

### Pre- and postsurvey comparison

Prior to engaging in the simulated HoloAnatomy classroom experience, the 21 medical students were asked 13 Likert-style questions about themselves and their experiences with and perceptions of AR. Four of these prestudy questions (7, 8, 9, and 10) correspond to those questions asked in the postsurvey (10, 11, 13, 14).

**FIG. 5.**
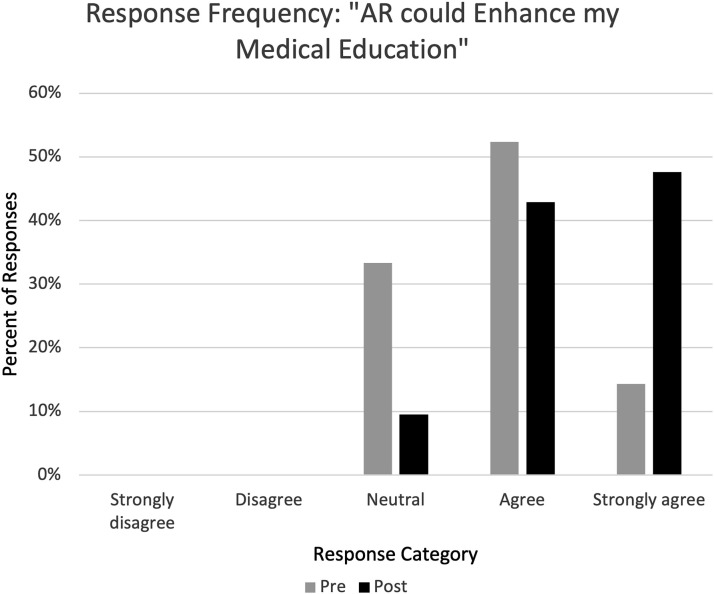
Percent category of pre- and poststudy participation survey responses to the statement, “AR could enhance my medical education.” No respondents replied as “strongly disagree” or disagree.” AR, augmented reality.

When asked in the prestudy survey whether students believed that AR technology could enhance their medical education, 67% (*n* = 14) responded as agreeing or strongly agreeing, 33% (*n* = 7) responded neutrally, and 0% (*n* = 0) responded as disagreeing or strongly disagreeing (Table 2). When students were asked again in the poststudy survey, 90% (*n* = 19) responded as agreeing or strongly agreeing, 10% (*n* = 2) responded neutrally, and 0% (*n* = 0) responded as disagreeing or strongly disagreeing (Table 3) (Fig. 5). This represented a statistically significant change in the perceptions toward AR technology regarding its potential in medical education overall (*p* < 0.001).

**Table 2.** Presurvey Responses Stratified by Question Identification

Statement	Positive	Neutral	Negative	Mode	Median
	***n* (%)**	***n* (%)**	***n* (%)**		
7. AR technology could enhance my medical educations.	14 (67)	7 (33)	0 (0)	4	4
8. Using AR technology would enhance my anatomy knowledge.	15 (71)	6 (29)	0 (0)	4	4
9. AR technology could be useful to better prepare me for my clinical rotations such as sim lab training.	14 (67)	6 (29)	1 (5)	4	4
10. AR technology would be useful for clinical treatment and therapy? (i.e., rehab, anxiety, pain control).	11 (52)	8 (38)	2 (10)	3	4

Median and mode were calculated using 1 = strongly disagree, 2 = disagree, 3 = neutral, 4 = agree, 5 = strongly agree.

**Table 3.** Postsurvey Responses Stratified by Question Identification

Statement	Positive	Neutral	Negative	Mode	Median
	***n* (%)**	***n* (%)**	***n* (%)**		
1. I want to learn more about AR technology.	19 (90)	2 (10)	0 (0)	4	4
2. The AR technology is difficult to learn and use.	14 (67)	4 (19)	13 (62)	2	2
3. I feel more familiar with AR technology.	21 (100)	0 (0)	0 (0)	5	5
4. The headset was comfortable to use.	14 (67)	5 (24)	2 (10)	4	4
5. The headset made me nauseous.	3 (14)	3 (14)	15 (71)	1	2
6. I found the AR image quality to be adequate.	12 (57)	6 (29)	3 (14)	4	4
7. I would like more experience with an AR headset.	19 (90)	2 (10)	0 (0)	4	4
8. I do not believe the AR technology is reliable for medical use.	3 (14)	3 (14)	15 (71)	2	2
10. I can see AR technology enhancing my medical education.	19 (90)	2 (10)	0 (0)	5	4
11. I can see AR technology improving my anatomy knowledge.	18 (86)	3 (14)	0 (0)	5	4
12. I would use AR technology over cadavers for anatomy education.	2 (10)	4 (19)	15 (71)	2	2
13. AR technology could be useful to better prepare me for my clinical rotations such as sim lab training.	14 (67)	4 (19)	3 (14)	4	4
14. AR technology would be useful for clinical treatment and therapy? (i.e., rehab, anxiety, pain control).	13 (62)	4 (19)	4 (19)	4	4

Median and mode were calculated using 1 = strongly disagree, 2 = disagree, 3 = neutral, 4 = agree, 5 = strongly agree.

**FIG. 6.**
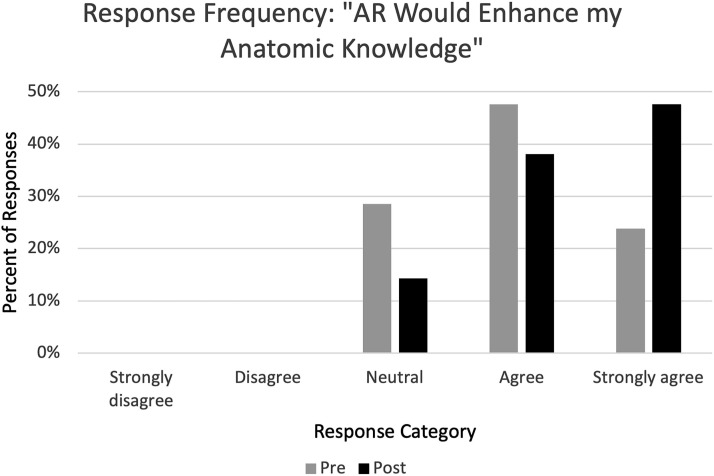
Percent category of pre- and poststudy participation survey responses to the statement, “AR would enhance my anatomical knowledge.” No respondents replied as “strongly disagree” or disagree.”

With regards to anatomy knowledge acquisition, when asked if using AR technology would enhance their anatomy knowledge, 71% (*n* = 15) of students responded as agreeing or strongly agreeing, 29% (*n* = 6) responded neutrally, and 0% (*n* = 0) responded as disagreeing or strongly disagreeing (Table 2). When asked again in the poststudy survey, 86% (*n* = 18) of students responded as agreeing or strongly agreeing, 14% (*n* = 3) responded neutrally, and 0% (*n* = 0) responded as disagreeing or strongly disagreeing (Table 3) (Fig. 6). This represented a statistically significant change in students’ perceptions toward AR technology regarding its potential in anatomy knowledge acquisition (*p* = 0.029).

When asked in the prestudy survey about AR’s usefulness to better prepare students for their clinical rotations such as sim lab training, 67% (*n* = 14) of students responded as agreeing or strongly agreeing, 29% (*n* = 6) responded neutrally, and 5% (*n* = 1) responded as disagreeing or strongly disagreeing (Table 2). In the postsurvey when asked again about the utility of AR in relation to clinical rotations, 67% (*n* = 14) of students responded as agreeing or strongly agreeing, 19% (*n* = 4) responded neutrally, and 14% (*n* = 3) responded as disagreeing or strongly disagreeing (Table 3) (Supplementary Fig. S1). This result was not statistically significant (*p* = 0.384), although it is important to note that while some participants viewed the technology more favorably in this regard after participation, an equal number viewed it less favorably after participation.

Lastly, when asked on the presurvey if AR technology would be useful for clinical treatment and therapy (i.e., rehab, anxiety, pain control), 52% (*n* = 11) of students responded as agreeing or strongly agreeing, 38% (*n* = 8) responded neutrally, and 10% (*n* = 2) responded as disagreeing or strongly disagreeing (Table 2). On the postsurvey, 62% (*n* = 13) of students responded as agreeing or strongly agreeing, 19% (*n* = 4) responded neutrally, and 19% (*n* = 4) responded as disagreeing or strongly disagreeing (Table 3) (Supplementary Fig. S2). This result was not statistically significant (*p* = 0.545).

## Discussion

This study demonstrated the potential application of AR technology to enhance medical education curricula. Although 48% of participants initially felt unfamiliar with AR, an overwhelming number of participants (90%) expressed that they could envision AR technology enhancing their medical education postintervention. This represents a potential area of untapped pedagogical growth. The feasibility of AR as a supplement to medical schools’ curricula is further supported by a statistically significant improvement in its perceptions among medical students when assessed before and after exposure to the teaching modules.

AR may soon join digital textbooks and computerized notebooks as a mainstay for educating medical students. In a review of AR’s potential as a medical educational technology, Kamphuis et al. asserted that “educational technology has the potential to offer a safe, suitable, and cost-effective training.”^4^

Within the field of medical education, AR offers unique advantages, specifically with regard to teaching students gross anatomy when compared to using traditional cadaveric dissection. Currently, the majority of medical schools across the globe depend heavily on cadaveric dissection labs for their anatomy and physiology training programs. Cadavers are challenging to obtain; a broad literature review published in 2018 identified that most countries rely on unclaimed bodies and the bodies of executed prisoners for cadaveric dissection labs in medical classrooms, without their consent.^8^ To counteract this, some schools utilize an ethical cadaver donation program. Yet, this presents an additional challenge of cost. In an interview with National Geographic, the Director of Anatomy at the University of Nevada, Las Vegas School of Medicine, Jeffrey Fahl, reported that “to build a cadaver lab that met government safety and health regulations would have cost about $10 million.” In the same article, the University of Colorado School of Medicine reported spending $1,900 per cadaver, of which they have ∼24 at any given time.^9^ With these aforementioned challenges, student-to-cadaver ratios as high as 8:1 can be found at some institutions.^10^ This can result in issues of disparity of access and engagement for a diverse student body in which not all students learn in the same way.

Further drawbacks to cadaver labs include the fact that many preservation methods use formalin, a formaldehyde-containing compound. Formaldehyde is a known carcinogen and a strong irritant. Long-term health impacts on faculty and students within gross-dissection labs have been incompletely characterized.^11^ Another vulnerability was shown during the COVID-19 pandemic, with medical schools worldwide reporting shortages of cadavers that met safety parameters.

With these disadvantages of cadaveric dissection in mind, the advantages of AR become more apparent. Given increasing access to—and decreasing costs of—AR technology, it is now feasible for medical schools to consider purchasing individual headsets for students. Such was the case at CWRU SOM. This approach sought to potentially address the dilemma of different personalities and learning styles, as the student-to-AR glasses ratio is 1:1 in comparison to the student-to-cadaver ratio, which is upwards of 8:1. With this, one may assume the collaborative aspect is reduced. However, collaboration between students could, in fact, be enhanced using AR as students retain the ability to work in small groups anytime, anywhere through AR collaborative learning sessions. These aspirations must be tempered, however, given a nonhomogenous agreement of AR ease of use—specifically highlighted in this study as 62% of respondents still found AR difficult to use after participating. Much of this sentiment likely stems from the gesture controls used by the software. During the sessions, participants often encountered instances where the goggles were not flawlessly detecting their inputs. At times, this made accessing the desired functionality clunky. This was likely compounded by the relative novelty of the HoloLens’ interface and controls, even to those who had used other AR goggles before. Similarly, not all participants agreed that AR would better prepare them for clinical rotations. This leaves the question of what limitations participants saw to the use of AR in this—a key realm of medical andragogy. Possible limitations include technical difficulties such as inexperience with the use of AR or a preference for more traditional means of learning.

Moreover, the role of AR in clinical training takes on a new dimension as it enables the practice of high acuity skills in a safe environment as described by Kamphius et al. when they note that “learners can make errors without adverse consequences, while instructors can focus on learners rather than patients.”^4^ AR dissections also offer personalized learning on individual models, 24 h a day, an advantage over a resource-intensive and supply-limited cadaver dissection lab. In a peri-COVID-19 world, these AR learning platforms are resistant to local guidelines and shifting geopolitical landscapes, further contributing to their value within medical education.

While AR hardware is not inexpensive (e.g., the Microsoft HoloLens 2 costs ∼$4,000), proponents of the technology argue that its value can be realized through its reusability. Headsets are passed down from class to class which can potentially offset initial investment to some degree. Additionally, medical schools often rotate through rotations at different times of the year. This would mean that for a class of, for example, 100 students, an institution would not need to purchase 100 headsets but rather, fewer. Perhaps only 25 or so. Further, as manufacturing improves and technology advances, it is likely that the unit price of headsets will decrease further. Additionally, the AR modules used for teaching are highly customizable, allowing for improvements alongside medical curriculum changes.

This study has several potential limitations, including a small participant size drawn from a single medical institution. Prior exposure to AR/VR headsets was not controlled for, leaving room for potentially confounding previous experiences. Additionally, the study was survey-based, assessing only students’ perceptions and impressions surrounding the technology. Students’ clinical knowledge acquisition was not tested in order to determine if there was a change between pre- and postintervention. To gauge knowledge acquisition, it would be important to incorporate knowledge-based questions in addition to the Likert-style questions related to students’ perceptions. In future investigations, there is a plan to test knowledge acquisition as a result of AR technology use. In addition, a future aim includes incorporating a direct clinical use case as a way of authentically assessing AR’s impact on students’ clinical skills. In order to expand these results, a future, larger multisite study would be beneficial. It would also be worthwhile to compare different commercially available AR hardware in order to determine the optimal standards for medical training use.

## Conclusion

This study found that less than half of the respondents were familiar with AR technology and its potential role within medical education prior to this exposure. Moreover, there was a significant shift in students’ perceptions, with most students reporting that AR technology could improve anatomy knowledge acquisition and enhance medical education overall. These findings suggest the importance of increasing both awareness of and understanding of AR within medical education so that it can be appropriately adopted and utilized to move training forward.

## Data Availability

The datasets generated and analyzed during this study are available from the corresponding author upon reasonable request.

## Abbreviations Used

AR: augmented reality
CWRU SOM: Case Western Reserve University School of Medicine
QR code: quick response code
VR: virtual reality
